# ER Dynamics and Derangement in Neurological Diseases

**DOI:** 10.3389/fnins.2018.00091

**Published:** 2018-02-20

**Authors:** Tomoyuki Yamanaka, Nobuyuki Nukina

**Affiliations:** Laboratory of Structural Neuropathology, Graduate School of Brain Science, Doshisha University, Kyoto, Japan

**Keywords:** endoplasmic reticulum, ER architecture, ER dynamics, neurological disease, neurodegeneration, NF-Y

## Abstract

The endoplasmic reticulum (ER) is a morphologically dynamic organelle containing different membrane subdomains with distinct cellular functions. Numerous observations have revealed that ER stress response induced by disturbed ER homeostasis is linked to various neurological/neurodegenerative disorders. In contrast, recent findings unveil that ER structural derangements are linked to the progression of several neurological diseases. The derangements involve two distinct, and likely opposing pathways. One is dysfunction of ER dynamics machinery, leading to disruption of ER network organization. Another one is facilitation of pre-existing machinery, leading to generation of markedly-ordered *de novo* membranous structure. Restoring the ER network can be the effective way toward the cure of ER-deranged neurological disorders.

## Introduction

**Figure 1:**
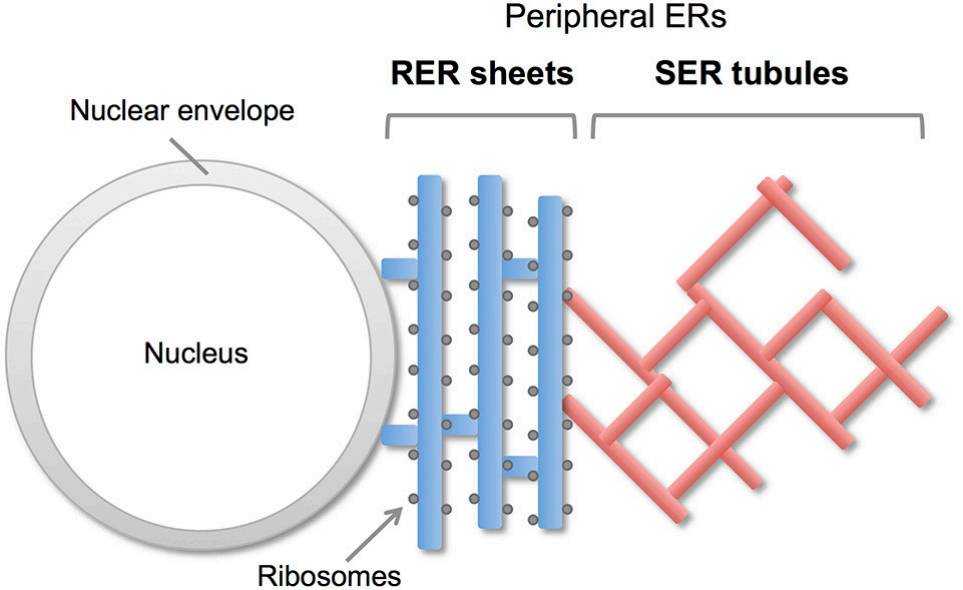
Morphology of peripheral ERs in mammalian cells. The peripheral ERs are composed of two different subdomains; a ribosome-studded rough ER (RER) and a ribosome-free smooth ER (SER). The RERs form sheets in perinuclear region and play a role in protein synthesis and transport. The SERs form tubule networks in cytoplasm and are involved in lipids synthesis, calcium storage and detoxification.

Endoplasmic reticulum (ER) is a continuous membrane organelle dispersing throughout the cells. It consist of two differentially shaped membranous domains; the nuclear envelope, a highly regulated membrane barrier that separates the nucleus from the cytoplasm, and the peripheral ERs including ribosome-studded rough ER (RER) and ribosome-free smooth ER (SER) (Figure 1). The RER plays a key role in synthesis and transport of secretary/membrane proteins. The SER is critical for synthesis of lipids / sterols, storage and regulated release of calcium, and metabolism and detoxification. These peripheral ERs are highly dynamic and change their shapes and volumes on the demand of cellular needs (Federovitch et al., 2005; Park and Blackstone, 2010; Westrate et al., 2015).

ER dysfunction causes deleterious effects on the cells and is associated with many diseases including various neurological/neurodegenerative disorders. The major pathway involved in this is ER stress responses induced by disturbance of ER homeostasis due to protein misfolding and aggregation (Matus et al., 2011; Remondelli and Renna, 2017). In contrast, recent findings highlight another ER abnormalities associated with ER morphological alteration in neurological diseases. These are mainly caused by dysregulations of ER-resident membrane proteins. In this review, we discussed the ER dynamics and its derangement in neuropathogenesis.

### Peripheral ER organization and dynamics

**Figure 2:**
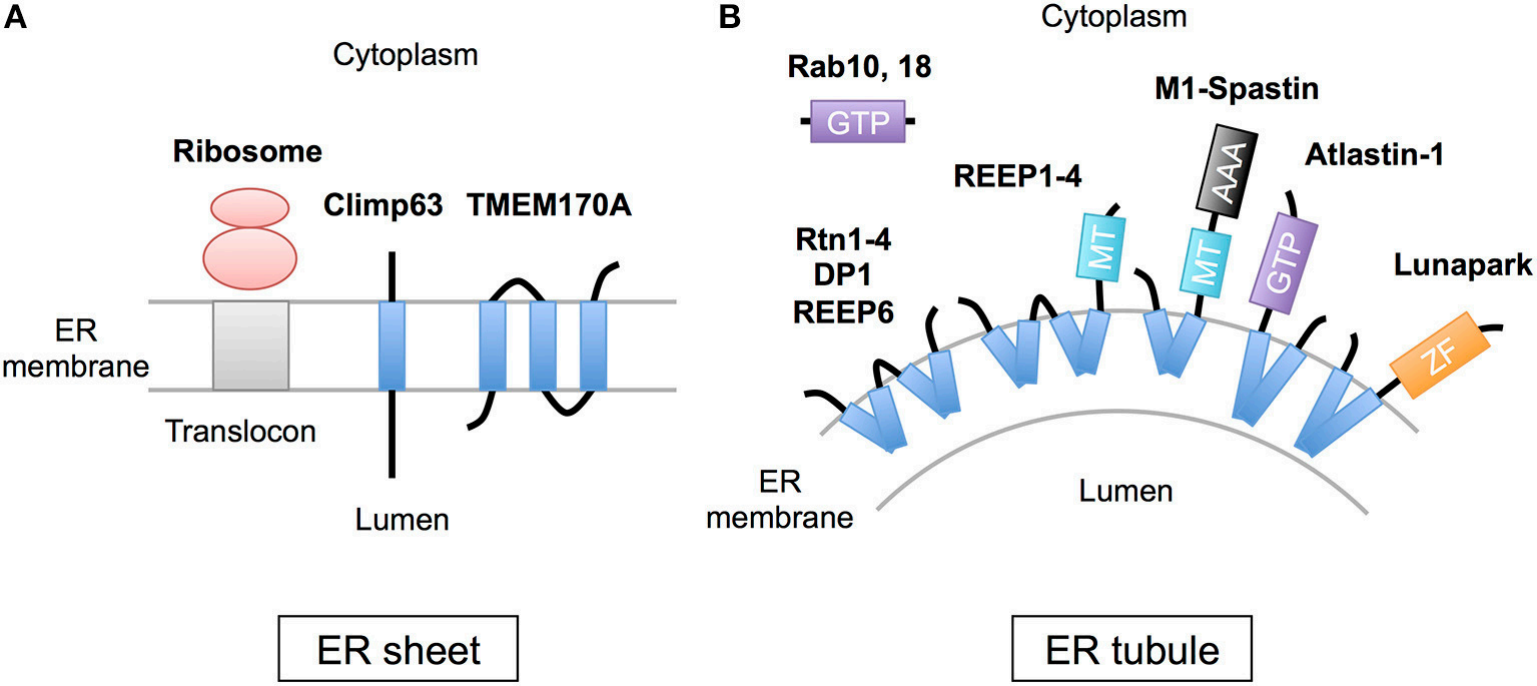
Scheme of the proteins regulating ER morphology. **(A)** The ER sheet formation is regulated by membrane-attaching ribosomes and two transmembrane proteins, Climp63 and TMEM170A. **(B)** The ER tubule formation is regulated by a set of hairpin transmembrane domain-containing proteins, including Reticulons (Rtn1-4), DP1 (REEP5), REEP1-4, REEP6, Atlastin-1, M1-Spastin and Lunapark, those generate curvature to bend the membrane. Rab GTPases (10 and 18) are also involved in this process. MT; microtubule binding region, GTP, GTPase domain; ZF, zinc finger motif.

The peripheral ERs are morphologically subdivided into two domains, ER sheets and tubules (Figure 1). The sheets mainly located in perinuclear region and tend to be studded with ribosomes (RER), whereas tubules form cytoplasmic network and are largely devoid of ribosomes (SER). Different sets of proteins are involved in the organization of these distinct ER membrane domains (Figure 2; Park and Blackstone, 2010; Chen et al., 2013; Westrate et al., 2015). In the sheets, one of the key regulators is stubbed ribosome, which is elegantly shown by the experiment using two translation inhibitors, puromycin or cycloheximide; the ER sheets are disrupted by puromycin that quickly dissociates polysomes from ER membranes, but not by cycloheximide that stabilizes ribosomal association with translocons, in cultured cells (Puhka et al., 2007). Two transmembrane protein Climp63 and TMEM170A are also shown to be important regulators for RER sheet formation; knockdown of each one decreased ER sheets whereas its overexpression increased them (Shibata et al., 2010; Christodoulou et al., 2016). As for TMEM170A, its localization to nuclear envelopes and regulation of nuclear morphology are also reported (Christodoulou et al., 2016). Thus, the ER sheets are organized by different types of proteins (Figure 2).

On the contrary, the ER tubules are generated by a set of specific membrane proteins that contain one or two “hairpin” transmembrane domains. This domain is inserted into outer leaflet of the membrane bilayer and suggested to generate curvature to bend the membrane (Figure 2; Park and Blackstone, 2010; Chen et al., 2013; Westrate et al., 2015). The membrane proteins include Reticulons (Rtn1-4), DP1 (deleted in polyposis locus 1; also known as REEP5), other REEPs (receptor expression-enhancing proteins; REEP1-4 and 6), Atlastin-1, M1-Spastin and Lunapark. Among them, Reticulons, DP1 and Atlastin-1 are extensively analyzed and shown to be required for formation of ER tubular network *in vitro* (Hu et al., 2008; Wang et al., 2016; Powers et al., 2017) and in cultured cells (Voeltz et al., 2006; Hu et al., 2009). These three proteins interact with each other (Hu et al., 2009), and form immobile oligomer complex in the tubules (Shibata et al., 2008; Orso et al., 2009). Complex formation of REEP1 with Atlastin-1 and M1-Spastin and its contribution of ER tubule formation are also reported (Evans et al., 2006; Park et al., 2010). Thus, interconnected interactions of these membrane-inserting proteins mediate the ER tubule formation. Atlastin-1 and Lunapark also induce homotypic ER fusion to generate branched tubules (Orso et al., 2009; Chen et al., 2012, 2015). Binding to microtubule cytoskeleton through REEP1-4 and M1-spastin is further involved in establishment and maintenance of complicated tubular network (Park and Blackstone, 2010; Westrate et al., 2015).

### Disruption of ER dynamics in neurological diseases

Notably, some of the ER tubule proteins have been shown to be mutated in hereditary spastic paraplegia (HSP), a group of genetic disorders caused by a length-dependent, distal axonopathy of corticospinal motor neurons, leading to lower limb spasticity and weakness. Currently, over 40 different genetic loci (SPG1–45) are reported (Blackstone et al., 2011; Lo Giudice et al., 2014). Despite the large number of loci, about 60% of HSP patients harbor pathogenic mutations in one of three proteins: Spastin (SPG4), Atlastin-1 (SPG3A), or REEP1 (SPG31), all of which are the regulators of ER tubule formation as described above. In addition, another ER tubule protein Rtn2 is also mutated in SPG12 patients. Thus, these SPG proteins are critical for motor neuron integrity *in vivo*. Indeed, knockdown of Spastin or Atlastin-1 in zebrafish induces axonal degeneration in motor neurons and locomotion defects (Butler et al., 2010; Fassier et al., 2010; Patten et al., 2014). In mice, mutation in Spastin or REEP1 causes progressive axonal degeneration of corticospinal motor neurons and motor defects (Tarrade et al., 2006; Beetz et al., 2013; Renvoise et al., 2016). Furthermore, peripheral ER complexity decreases in primary motor neurons of REEP1 mutant mice (Beetz et al., 2013). These observations suggest that these SPG proteins are necessary for axonal development and/or maintenance probably by modulating ER architecture in motor neurons. Because Spastin, Atlastin-1, and REEP1 are co-enriched in axonal growth cones in cultured neurons (Park et al., 2010), they may regulate axonal growth by modulating ER tubule network dynamics.

The other factors involved in the ER tubule formation are Rab GTPases, central regulators of vesicle budding, motility, and fusion (Figure 2). Two Rab GTPases, Rab10, and Rab18, are shown to localize to ER tubules and its dysfunction results in ER tubule disorganization in cultured cells (English and Voeltz, 2013; Gerondopoulos et al., 2014). Notably, loss-of-function mutations of Rab18 or its regulator RabGAP cause Warburg Micro Syndrome, a rare autosomal recessive genetic disorder characterized by severe eye and brain abnormalities (Bem et al., 2011). Knockdown of Rab18 in zebrafish causes developmental abnormalities including microphthalmia and microcephaly (Bem et al., 2011). In addition, knockdown of Rab10 or Rab18 induces defects in neuronal differentiation in mouse brain cortex (Wang et al., 2011; Wu et al., 2016). These observations reveal critical roles of these Rab GTPases in neuronal development *in vivo*.

An ER membrane protein FAM134B is also linked to a neurological disease named hereditary sensory and autonomic neuropathy (HSAN) (Kurth et al., 2009). Interestingly, this protein is shown to be an autophagy receptor to mediate degradation of ER through autophagic system. This phenomenon is called ER-phagy, which is considered to be involved in ER homeostasis (Khaminets et al., 2015). Being its localization mainly to ER sheets, FAM134B is thought to be an ER sheet-specific autophagy receptor. Recently, an ER tubule protein Rtn3 is identified as another ER-phagy receptor, which induce fragmentation and autophagic degradation of ER tubules independently of FAM134B (Grumati et al., 2017). Thus, FAM134B and Rtn3 may regulate different ER membranes to regulate overall ER homeostasis.

### The ER tubule network and neurodegenerative diseases

The ER tubules are interacting with other membranous organelles such as mitochondria, plasma membrane, peroxisomes and lysosomes. The mitochondria-associated ER membrane (MAM) mediates several fundamental cellular processes including calcium exchange, phospholipid exchange, intracellular trafficking and autophagy. The ER–mitochondria associations through MAM are shown to be disrupted in several neurodegenerative diseases such as Alzheimer's disease (AD), Parkinson's disease and amyotrophic lateral sclerosis with associated frontotemporal dementia (ALS/FTD), resulting in alteration in cellular functions regulated by MAM (Paillusson et al., 2016).

Direct implications of ER tubule proteins in disease pathogenesis have been reported. In superoxide dismutase 1 (SOD1) G93A transgenic mouse, a model for ALS, depletion of Rtn4 accelerates disease onset and progression possibly by disrupting normal distribution of protein disulfide isomerase (PDI), suggesting protective role of Reticulon in motor neuron degeneration (Yang et al., 2009). As for AD, Rtn3 binds to and colocalizes with BACE1, a beta-secretase involved in amyloid precursor protein (APP) cleavage and amyloid beta production. Interestingly, overexpression of Rtn3 reduces the production of amyloid beta while its knockdown enhances it in cultured cells. Furthermore, Rtn3 blocks BACE1 interactions with APP, suggesting that the Reticulon negatively modulates BACE1 activity and amyloid beta production (He et al., 2004). In addition, enhanced expression of Rtn3 suppresses amyloid plaque formation in transgenic mice expressing mutants for APP and presenilin-1 (Shi et al., 2009). In contrast to these beneficial effects, Rtn3 is found to be aggregated and accumulated in dystrophic neurites, named as Rtn3 immunoreactive dystrophic neurites (RIDNs) in brains of AD cases and mice brains expressing mutant APP. Furthermore, Rtn3 transgenic expression impairs spatial learning and memory as well as synaptic plasticity in mice, implying that RIDNs potentially contribute to AD cognitive dysfunction (Hu et al., 2007). Thus, Rtn3 may bidirectionally regulate AD pathogenesis *in vivo*.

### Biogenesis of highly-ordered ERs, the stacked SERs

In addition to the tubular architecture as described above, the SER is known to form highly-organized membranous structures where cisternae are stacked with ordered arrays (Federovitch et al., 2005; Borgese et al., 2006). This “stacked SER” is observed in the cells highly demanding SER-related functions such as lipid biosynthesis and drug metabolism. These include adrenal cells that produce large amount of sterol lipids, and liver cells of animal treated with phenobarbital (Feldman et al., 1981; Federovitch et al., 2005). Treatment of statins (cholesterol synthesis inhibitors) also leads to stacked SER formation by induced expression of an ER-resident enzyme, hydroxy-methylglutaryl (HMG)-CoA reductase (Singer et al., 1988; Borgese et al., 2006). Interestingly, overexpression of ER resident membrane proteins such as cytochrome b(5), P450, aldehyde dehydrogenase, Sec61 and Calnexin in cultured cells is shown to be sufficient for stacked SER formation (Yamamoto et al., 1996; Snapp et al., 2003; Korkhov and Zuber, 2009). Thus, load of ER-resident membrane proteins is one of the factors for stacked SER biogenesis.

As for the molecular mechanism, selective activation of ATF6 and following lipid synthesis are suggested to be involved in stacked SER generation upon cytochrome b(5) expression (Maiuolo et al., 2011). Because of no inductions of ER chaperone expression, XBP1 splicing or eIF2-alpha phosphorylation, the usual unfolded protein response (UPR) pathway may not be involved in ATF6-dependent pathway. Knockdown of Syntaxin 18, a SNARE component involved in ER-Golgi transport and ER-network organization, also induces stacked SER formation (Iinuma et al., 2009). Furthermore, knockdown of a membrane protein Yip1A, which cycles between ER and early Golgi, induces formation of stacked SER (Dykstra et al., 2010). A compound phenyl-2-decanoyl-amino-3-morpholino-1-propanol-hydrocholride (PDMP) that blocks membrane transport from ER to Golgi also induces generation of this type of ER (Sprocati et al., 2006). Experiments using several compounds further suggested that altering ionic homeostasis in ER is also an inducer of stacked SER formation independently of known UPR pathways (Varadarajan et al., 2012, 2013). Notably, the stacked SER formation often accompanies Golgi fragmentation and delay of ER export (Iinuma et al., 2009; Dykstra et al., 2010; Varadarajan et al., 2012). These observations suggest close relationship of ER-Golgi transport machineries, rather than canonical UPR pathways, to stacked SER biogenesis.

Despite the drastic alterations of ER and Golgi architectures, there is no report describing distinct reduction of cell viability. Importantly, the ER structure is reversible because depletion of the compounds such as PDMP leads to disappearance of stacked SER (Sprocati et al., 2006; Varadarajan et al., 2012). Thus, the stacked SER formation may not be the toxic inducer for cells but is suggested to be a novel stress response to cope with overload of ER membrane proteins independently of usual UPR pathways.

### Stacked SER pathologies in neurological diseases

**Figure 3:**
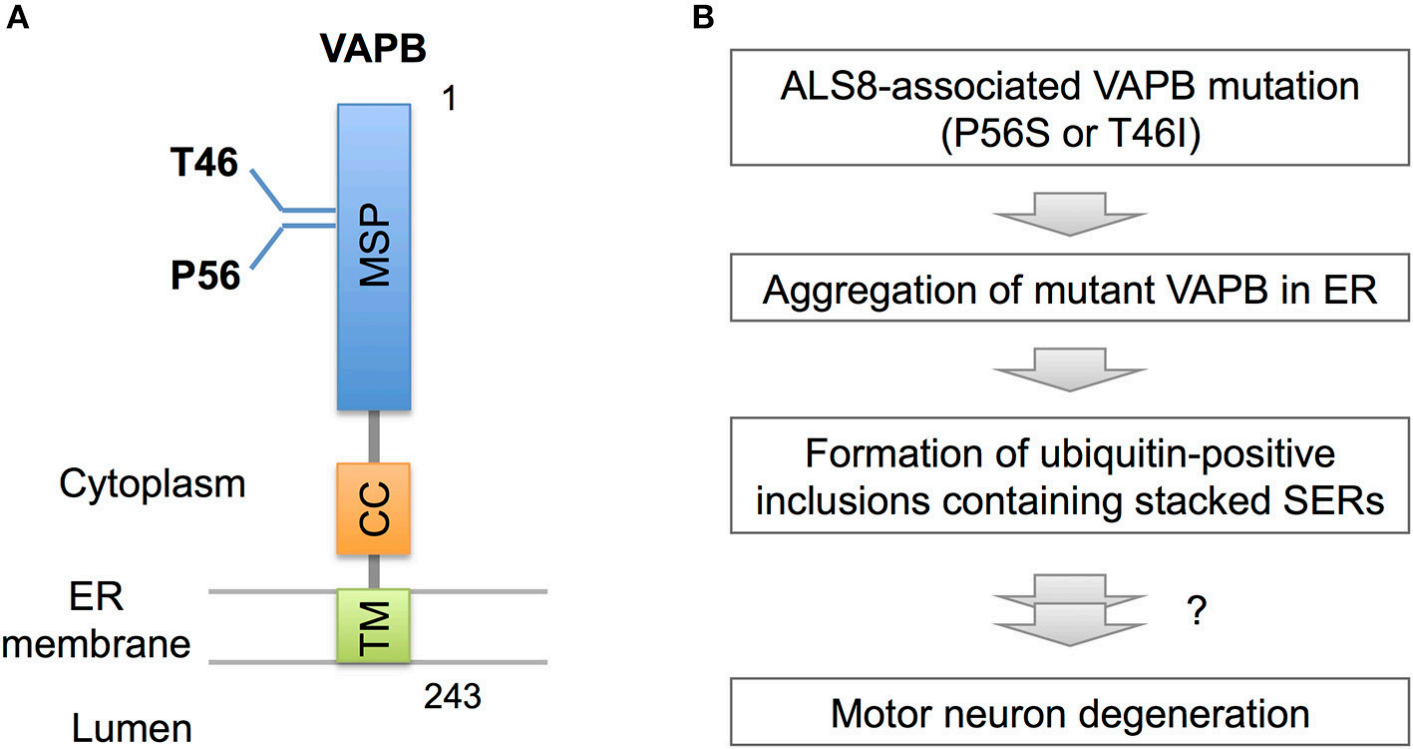
VAPB mutations and SER pathology. **(A)** Domain structure of VAPB. VAPB is composed of the 125-residue major sperm protein (MSP), coiled coil (CC), and transmembrane (TM) domains. The residues Thr46 and Pro56 are mutated to Ile and Ser, respectively, in familial ALS (ALS8). **(B)** A model for VAPB-pathology in ALS8. The mutant VAPB proteins are insolubilized and aggregated in ER, leading to formation of ubiquitin-positive inclusions containing stacked SER. The direct link of ER pathology to motor neuron degeneration is still uncertain, however.

Recent observations, however, indicate pathogenic significance of stacked SER in neurological diseases. In familial ALS (ALS8), an ER-resident membrane protein, vesicle-associated membrane protein-associated protein B (VAPB) is mutated (Figure 3; Nishimura et al., 2004). Overexpressed mutant VAPB protein in cultured cells is insolubilized and aggregated in ER, leading to formation of ubiquitin-positive inclusions containing stacked SERs (Figure 3; Teuling et al., 2007; Fasana et al., 2010). In cultured neurons, the mutant VAPB overexpression induces Golgi fragmentation and cell death (Teuling et al., 2007). Furthermore, in VAPB mutant-transgenic mice ubiquitin-positive inclusions associated with stacked SER are developed in motor neurons (Tudor et al., 2010; Aliaga et al., 2013; Kuijpers et al., 2013; Qiu et al., 2013), and furthermore progressive loss of corticospinal motor neurons is observed in some of mice lines (Aliaga et al., 2013). Although the neurotoxic effect of mutant VAPB is still controversial because other transgenic mice lines do not show neuronal loss or motor phenotypes (Tudor et al., 2010; Kuijpers et al., 2013; Qiu et al., 2013), VAPB mutant knock-in mice are shown to display slow progression of motor behavior defects (Larroquette et al., 2015), suggesting a certain involvement of VAPB mutation on motor neuron dysfunction.

Torsion dystonia-1 (DYT1) is the most common inherited dystonia characterized by involuntary muscle contractions and abnormal postures, which is caused by mutation in TorsinA, an ER glycoprotein belonging to AAA family of proteins (Ozelius et al., 1997). The mutant TorsinA forms cytoplasmic inclusions containing stacked SER in cultured neuronal cells (Hewett et al., 2000; Gonzalez-Alegre and Paulson, 2004). In peripheral nervous system, mutation in peripheral myelin protein 22 (PMP22) is linked to Charcot-Marie-Tooth disease, a sensorineural peripheral polyneuropathy. PMP22 is expressed in Schwann cells and its mutation induces formation of cytoplasmic inclusion containing mutant protein in association with stacked SER formation (Dickson et al., 2002). These observations further provide the pathological significance of cytoplasmic inclusions containing stacked SER in neurological diseases.

**Figure 4:**
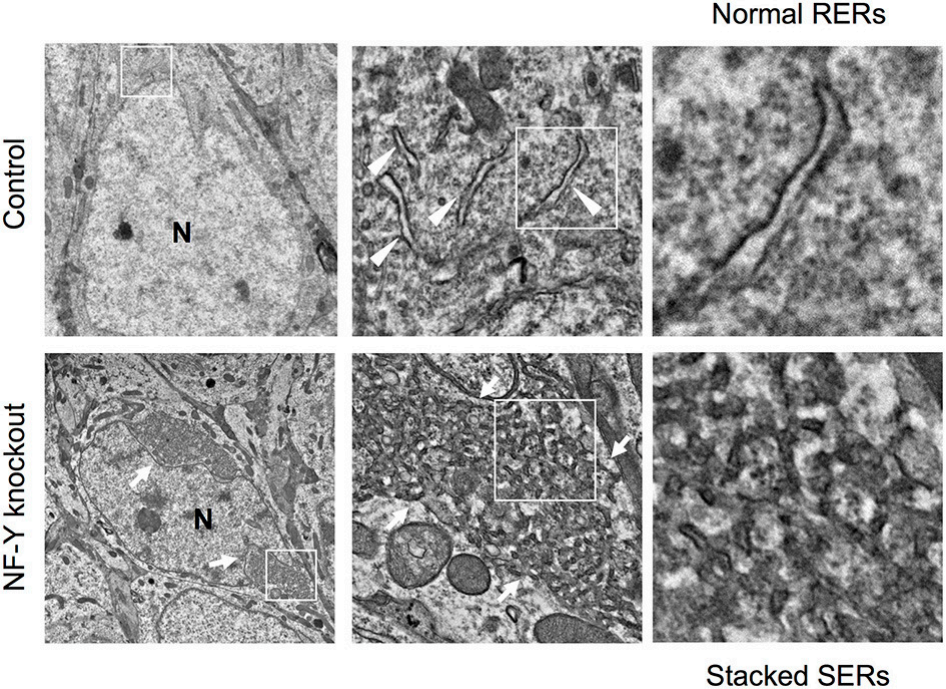
SER pathology in NF-Y knockout neurons. Electron microgram of hippocampal CA1 neurons in control and NF-Y knockout mouse. Boxed areas are enlarged in the next panels. The neurons of NF-Y knockout mouse contain stacked SERs at perinuclear regions (arrows in lower panels) whereas control mouse neurons contain normal RERs (arrowheads in upper panels). N indicates nucleus. The data are reproduced from our previous paper (Yamanaka et al., 2014) with slight modifications.

A novel mouse model for stacked SER pathology in brain neurons was recently established (Yamanaka et al., 2014, 2016). Importantly, it does not involve disease-associated gene mutation but just caused by inactivation of CCAAT-binding factor NF-Y, a ubiquitous transcription factor shown to be affected in polyglutamine diseases (Yamanaka et al., 2008; Katsuno et al., 2010; Huang et al., 2011). The neuron-specific knockdown of NF-Y induces insolubilization of various membrane proteins including Calnexin, Reticulon, Atlastin-1, APP and Carboxypeptidase E together with ubiquitin and p62/Sqstm1, all of which are accumulated on ER (Yamanaka et al., 2014, 2016). It also accompanies perinuclear accumulation of ribosome-free SERs and Golgi disassembly (Figure 4). Chromatin immunoprecipitation identifies several genes involved in protein folding in ER and ER-associated degradation (ERAD) as targets of NF-Y, suggesting a critical role of NF-Y in ER protein homeostasis to maintain normal ER architecture in brain neurons.

## Conclusions

Among the peripheral ERs, SERs appear to be deranged in several neurological diseases through at least two pathways. One is disturbance of peripheral ER network in the diseases such as HSPs, which is caused by mutations of genes required for SER tubule organization. The SER network disruption may also be involved in Alzheimer's disease and ALS pathogenesis. Another one is development of highly-ordered stacked SER in the diseases such as ALS8, which is caused by overload of misfolded and insolubilized proteins on ER membranes. NF-Y-mediated gene regulatory pathway can be involved in this atypical ER pathogenesis. Finding the way to restore SER networks is a future challenging issue toward the cure of these ER-related neurological disorders.

## Author contributions

All authors listed have made a substantial, direct and intellectual contribution to the work, and approved it for publication.

### Conflict of interest statement

The authors declare that the research was conducted in the absence of any commercial or financial relationships that could be construed as a potential conflict of interest.

## References

[B1] Aliaga L. Lai C. Yu J. Chub N. Shim H. Sun L.. (2013). Amyotrophic lateral sclerosis-related VAPB P56S mutation differentially affects the function and survival of corticospinal and spinal motor neurons. Hum. Mol. Genet. 22, 4293–4305. 10.1093/hmg/ddt279 23771029 PMC3792689

[B2] Beetz C. Koch N. Khundadze M. Zimmer G. Nietzsche S. Hertel N.. (2013). A spastic paraplegia mouse model reveals REEP1-dependent ER shaping. J. Clin. Invest. 123, 4273–4282. 10.1172/JCI65665 24051375 PMC3784524

[B3] Bem D. Yoshimura S. Nunes-Bastos R. Bond F. C. Kurian M. A. Rahman F.. (2011). Loss-of-function mutations in RAB18 cause Warburg micro syndrome. Am. J. Hum. Genet. 88, 499–507. 10.1016/j.ajhg.2011.03.012 21473985 PMC3071920

[B4] Blackstone C. O'kane C. J. Reid E. (2011). Hereditary spastic paraplegias: membrane traffic and the motor pathway. Nat. Rev. Neurosci. 12, 31–42. 10.1038/nrn2946 21139634 PMC5584382

[B5] Borgese N. Francolini M. Snapp E. (2006). Endoplasmic reticulum architecture: structures in flux. Curr. Opin. Cell Biol. 18, 358–364. 10.1016/j.ceb.2006.06.008 16806883 PMC4264046

[B6] Butler R. Wood J. D. Landers J. A. Cunliffe V. T. (2010). Genetic and chemical modulation of spastin-dependent axon outgrowth in zebrafish embryos indicates a role for impaired microtubule dynamics in hereditary spastic paraplegia. Dis. Model. Mech. 3, 743–751. 10.1242/dmm.004002 20829563 PMC2965401

[B7] Chen S. Desai T. McNew J. A. Gerard P. Novick P. J. Ferro-Novick S. (2015). Lunapark stabilizes nascent three-way junctions in the endoplasmic reticulum. Proc. Natl. Acad. Sci. U.S.A. 112, 418–423. 10.1073/pnas.1423026112 25548161 PMC4299238

[B8] Chen S. Novick P. Ferro-Novick S. (2012). ER network formation requires a balance of the dynamin-like GTPase Sey1p and the Lunapark family member Lnp1p. Nat. Cell Biol. 14, 707–716. 10.1038/ncb2523 22729086 PMC3389217

[B9] Chen S. Novick P. Ferro-Novick S. (2013). ER structure and function. Curr. Opin. Cell Biol. 25, 428–433. 10.1016/j.ceb.2013.02.006 23478217 PMC5614462

[B10] Christodoulou A. Santarella-Mellwig R. Santama N. Mattaj I. W. (2016). Transmembrane protein TMEM170A is a newly discovered regulator of ER and nuclear envelope morphogenesis in human cells. J. Cell Sci. 129, 1552–1565. 10.1242/jcs.175273 26906412 PMC4852765

[B11] Dickson K. M. Bergeron J. J. Shames I. Colby J. Nguyen D. T. Chevet E.. (2002). Association of calnexin with mutant peripheral myelin protein-22 *ex vivo*: a basis for “gain-of-function” ER diseases. Proc. Natl. Acad. Sci. U.S.A. 99, 9852–9857. 10.1073/pnas.152621799 12119418 PMC125041

[B12] Dykstra K. M. Pokusa J. E. Suhan J. Lee T. H. (2010). Yip1A structures the mammalian endoplasmic reticulum. Mol. Biol. Cell 21, 1556–1568. 10.1091/mbc.E09-12-1002 20237155 PMC2861614

[B13] English A. R. Voeltz G. K. (2013). Rab10 GTPase regulates ER dynamics and morphology. Nat. Cell Biol. 15, 169–178. 10.1038/ncb2647 23263280 PMC3582403

[B14] Evans K. Keller C. Pavur K. Glasgow K. Conn B. Lauring B. (2006). Interaction of two hereditary spastic paraplegia gene products, spastin and atlastin, suggests a common pathway for axonal maintenance. Proc. Natl. Acad. Sci. U.S.A. 103, 10666–10671. 10.1073/pnas.0510863103 16815977 PMC1502289

[B15] Fasana E. Fossati M. Ruggiano A. Brambillasca S. Hoogenraad C. C. Navone F.. (2010). A VAPB mutant linked to amyotrophic lateral sclerosis generates a novel form of organized smooth endoplasmic reticulum. FASEB J. 24, 1419–1430. 10.1096/fj.09-147850 20008544

[B16] Fassier C. Hutt J. A. Scholpp S. Lumsden A. Giros B. Nothias F.. (2010). Zebrafish atlastin controls motility and spinal motor axon architecture via inhibition of the BMP pathway. Nat. Neurosci. 13, 1380–1387. 10.1038/nn.2662 20935645

[B17] Federovitch C. M. Ron D. Hampton R. Y. (2005). The dynamic ER: experimental approaches and current questions. Curr. Opin. Cell Biol. 17, 409–414. 10.1016/j.ceb.2005.06.010 15975777

[B18] Feldman D. Swarm R. L. Becker J. (1981). Ultrastructural study of rat liver and liver neoplasms after long-term treatment with phenobarbital. Cancer Res. 41, 2151–2162. 7237417

[B19] Gerondopoulos A. Bastos R. N. Yoshimura S. Anderson R. Carpanini S. Aligianis I.. (2014). Rab18 and a Rab18 GEF complex are required for normal ER structure. J. Cell Biol. 205, 707–720. 10.1083/jcb.201403026 24891604 PMC4050724

[B20] Gonzalez-Alegre P. Paulson H. L. (2004). Aberrant cellular behavior of mutant torsinA implicates nuclear envelope dysfunction in DYT1 dystonia. J. Neurosci. 24, 2593–2601. 10.1523/JNEUROSCI.4461-03.2004 15028751 PMC6729521

[B21] Grumati P. Morozzi G. Holper S. Mari M. Harwardt M. I. Yan R.. (2017). Full length RTN3 regulates turnover of tubular endoplasmic reticulum via selective autophagy. Elife 6:e25555 10.7554/eLife.25555 28617241 PMC5517149

[B22] He W. Lu Y. Qahwash I. Hu X. Y. Chang A. Yan R. (2004). Reticulon family members modulate BACE1 activity and amyloid-beta peptide generation. Nat. Med. 10, 959–965. 10.1038/nm1088 15286784

[B23] Hewett J. Gonzalez-Agosti C. Slater D. Ziefer P. Li S. Bergeron D.. (2000). Mutant torsinA, responsible for early-onset torsion dystonia, forms membrane inclusions in cultured neural cells. Hum. Mol. Genet. 9, 1403–1413. 10.1093/hmg/9.9.1403 10814722

[B24] Hu J. Shibata Y. Voss C. Shemesh T. Li Z. Coughlin M.. (2008). Membrane proteins of the endoplasmic reticulum induce high-curvature tubules. Science 319, 1247–1250. 10.1126/science.1153634 18309084

[B25] Hu J. Shibata Y. Zhu P. P. Voss C. Rismanchi N. Prinz W. A.. (2009). A class of dynamin-like GTPases involved in the generation of the tubular ER network. Cell 138, 549–561. 10.1016/j.cell.2009.05.025 19665976 PMC2746359

[B26] Hu X. Shi Q. Zhou X. He W. Yi H. Yin X.. (2007). Transgenic mice overexpressing reticulon 3 develop neuritic abnormalities. EMBO J. 26, 2755–2767. 10.1038/sj.emboj.7601707 17476306 PMC1888669

[B27] Huang S. Ling J. J. Yang S. Li X. J. Li S. (2011). Neuronal expression of TATA box-binding protein containing expanded polyglutamine in knock-in mice reduces chaperone protein response by impairing the function of nuclear factor-Y transcription factor. Brain 134, 1943–1958. 10.1093/brain/awr146 21705419 PMC3122377

[B28] Iinuma T. Aoki T. Arasaki K. Hirose H. Yamamoto A. Samata R.. (2009). Role of syntaxin 18 in the organization of endoplasmic reticulum subdomains. J. Cell Sci. 122, 1680–1690. 10.1242/jcs.036103 19401338

[B29] Katsuno M. Adachi H. Minamiyama M. Waza M. Doi H. Kondo N.. (2010). Disrupted transforming growth factor-beta signaling in spinal and bulbar muscular atrophy. J. Neurosci. 30, 5702–5712. 10.1523/JNEUROSCI.0388-10.2010 20410122 PMC6632356

[B30] Khaminets A. Heinrich T. Mari M. Grumati P. Huebner A. K. Akutsu M.. (2015). Regulation of endoplasmic reticulum turnover by selective autophagy. Nature 522, 354–358. 10.1038/nature14498 26040720

[B31] Korkhov V. M. Zuber B. (2009). Direct observation of molecular arrays in the organized smooth endoplasmic reticulum. BMC Cell Biol. 10:59. 10.1186/1471-2121-10-59 19703297 PMC2737311

[B32] Kuijpers M. Van Dis V. Haasdijk E. D. Harterink M. Vocking K. Post J. A.. (2013). Amyotrophic lateral sclerosis (ALS)-associated VAPB-P56S inclusions represent an ER quality control compartment. Acta Neuropathol. Commun. 1:24. 10.1186/2051-5960-1-24 24252306 PMC3893532

[B33] Kurth I. Pamminger T. Hennings J. C. Soehendra D. Huebner A. K. Rotthier A.. (2009). Mutations in FAM134B, encoding a newly identified Golgi protein, cause severe sensory and autonomic neuropathy. Nat. Genet. 41, 1179–1181. 10.1038/ng.464 19838196

[B34] Larroquette F. Seto L. Gaub P. L. Kamal B. Wallis D. Lariviere R.. (2015). Vapb/Amyotrophic lateral sclerosis 8 knock-in mice display slowly progressive motor behavior defects accompanying ER stress and autophagic response. Hum. Mol. Genet. 24, 6515–6529. 10.1093/hmg/ddv360 26362257 PMC4614709

[B35] Lo Giudice T. Lombardi F. Santorelli F. M. Kawarai T. Orlacchio A. (2014). Hereditary spastic paraplegia: clinical-genetic characteristics and evolving molecular mechanisms. Exp. Neurol. 261, 518–539. 10.1016/j.expneurol.2014.06.011 24954637

[B36] Maiuolo J. Bulotta S. Verderio C. Benfante R. Borgese N. (2011). Selective activation of the transcription factor ATF6 mediates endoplasmic reticulum proliferation triggered by a membrane protein. Proc. Natl. Acad. Sci. U.S.A. 108, 7832–7837. 10.1073/pnas.1101379108 21521793 PMC3093499

[B37] Matus S. Glimcher L. H. Hetz C. (2011). Protein folding stress in neurodegenerative diseases: a glimpse into the ER. Curr. Opin. Cell Biol. 23, 239–252. 10.1016/j.ceb.2011.01.003 21288706

[B38] Nishimura A. L. Mitne-Neto M. Silva H. C. Richieri-Costa A. Middleton S. Cascio D.. (2004). A mutation in the vesicle-trafficking protein VAPB causes late-onset spinal muscular atrophy and amyotrophic lateral sclerosis. Am. J. Hum. Genet. 75, 822–831. 10.1086/425287 15372378 PMC1182111

[B39] Orso G. Pendin D. Liu S. Tosetto J. Moss T. J. Faust J. E.. (2009). Homotypic fusion of ER membranes requires the dynamin-like GTPase atlastin. Nature 460, 978–983. 10.1038/nature08280 19633650

[B40] Ozelius L. J. Hewett J. W. Page C. E. Bressman S. B. Kramer P. L. Shalish C.. (1997). The early-onset torsion dystonia gene (DYT1) encodes an ATP-binding protein. Nat. Genet. 17, 40–48. 10.1038/ng0997-40 9288096

[B41] Paillusson S. Stoica R. Gomez-Suaga P. Lau D. H. Mueller S. Miller T.. (2016). There's something wrong with my MAM; the ER-Mitochondria axis and neurodegenerative diseases. Trends Neurosci. 39, 146–157. 10.1016/j.tins.2016.01.008 26899735 PMC4780428

[B42] Park S. H. Blackstone C. (2010). Further assembly required: construction and dynamics of the endoplasmic reticulum network. EMBO Rep. 11, 515–521. 10.1038/embor.2010.92 20559323 PMC2897125

[B43] Park S. H. Zhu P. P. Parker R. L. Blackstone C. (2010). Hereditary spastic paraplegia proteins REEP1, spastin, and atlastin-1 coordinate microtubule interactions with the tubular ER network. J. Clin. Invest. 120, 1097–1110. 10.1172/JCI40979 20200447 PMC2846052

[B44] Patten S. A. Armstrong G. A. Lissouba A. Kabashi E. Parker J. A. Drapeau P. (2014). Fishing for causes and cures of motor neuron disorders. Dis. Model. Mech. 7, 799–809. 10.1242/dmm.015719 24973750 PMC4073270

[B45] Powers R. E. Wang S. Liu T. Y. Rapoport T. A. (2017). Reconstitution of the tubular endoplasmic reticulum network with purified components. Nature 543, 257–260. 10.1038/nature21387 28225760 PMC5853125

[B46] Puhka M. Vihinen H. Joensuu M. Jokitalo E. (2007). Endoplasmic reticulum remains continuous and undergoes sheet-to-tubule transformation during cell division in mammalian cells. J. Cell Biol. 179, 895–909. 10.1083/jcb.200705112 18056408 PMC2099207

[B47] Qiu L. Qiao T. Beers M. Tan W. Wang H. Yang B.. (2013). Widespread aggregation of mutant VAPB associated with ALS does not cause motor neuron degeneration or modulate mutant SOD1 aggregation and toxicity in mice. Mol. Neurodegener. 8:1. 10.1186/1750-1326-8-1 23281774 PMC3538568

[B48] Remondelli P. Renna M. (2017). The Endoplasmic Reticulum unfolded protein response in neurodegenerative disorders and its potential therapeutic significance. Front. Mol. Neurosci. 10:187. 10.3389/fnmol.2017.00187 28670265 PMC5472670

[B49] Renvoise B. Malone B. Falgairolle M. Munasinghe J. Stadler J. Sibilla C.. (2016). Reep1 null mice reveal a converging role for hereditary spastic paraplegia proteins in lipid droplet regulation. Hum. Mol. Genet. 25, 5111–5125. 10.1093/hmg/ddw315 27638887 PMC6078631

[B50] Shi Q. Prior M. He W. Tang X. Hu X. Yan R. (2009). Reduced amyloid deposition in mice overexpressing RTN3 is adversely affected by preformed dystrophic neurites. J. Neurosci. 29, 9163–9173. 10.1523/JNEUROSCI.5741-08.2009 19625507 PMC2743151

[B51] Shibata Y. Shemesh T. Prinz W. A. Palazzo A. F. Kozlov M. M. Rapoport T. A. (2010). Mechanisms determining the morphology of the peripheral ER. Cell 143, 774–788. 10.1016/j.cell.2010.11.007 21111237 PMC3008339

[B52] Shibata Y. Voss C. Rist J. M. Hu J. Rapoport T. A. Prinz W. A.. (2008). The reticulon and DP1/Yop1p proteins form immobile oligomers in the tubular endoplasmic reticulum. J. Biol. Chem. 283, 18892–18904. 10.1074/jbc.M800986200 18442980 PMC2441541

[B53] Singer I.i. Scott S. Kazazis D. M. Huff J. W. (1988). Lovastatin, an inhibitor of cholesterol synthesis, induces hydroxymethylglutaryl-coenzyme a reductase directly on membranes of expanded smooth endoplasmic reticulum in rat hepatocytes. Proc. Natl. Acad. Sci. U.S.A. 85, 5264–5268. 10.1073/pnas.85.14.5264 3293052 PMC281730

[B54] Snapp E. L. Hegde R. S. Francolini M. Lombardo F. Colombo S. Pedrazzini E.. (2003). Formation of stacked ER cisternae by low affinity protein interactions. J. Cell Biol. 163, 257–269. 10.1083/jcb.200306020 14581454 PMC2173526

[B55] Sprocati T. Ronchi P. Raimondi A. Francolini M. Borgese N. (2006). Dynamic and reversible restructuring of the ER induced by PDMP in cultured cells. J. Cell Sci. 119, 3249–3260. 10.1242/jcs.03058 16847050

[B56] Tarrade A. Fassier C. Courageot S. Charvin D. Vitte J. Peris L.. (2006). A mutation of spastin is responsible for swellings and impairment of transport in a region of axon characterized by changes in microtubule composition. Hum. Mol. Genet. 15, 3544–3558. 10.1093/hmg/ddl431 17101632

[B57] Teuling E. Ahmed S. Haasdijk E. Demmers J. Steinmetz M. O. Akhmanova A.. (2007). Motor neuron disease-associated mutant vesicle-associated membrane protein-associated protein (VAP) B recruits wild-type VAPs into endoplasmic reticulum-derived tubular aggregates. J. Neurosci. 27, 9801–9815. 10.1523/JNEUROSCI.2661-07.2007 17804640 PMC6672975

[B58] Tudor E. L. Galtrey C. M. Perkinton M. S. Lau K. F. De Vos K. J. Mitchell J. C.. (2010). Amyotrophic lateral sclerosis mutant vesicle-associated membrane protein-associated protein-B transgenic mice develop TAR-DNA-binding protein-43 pathology. Neuroscience 167, 774–785. 10.1016/j.neuroscience.2010.02.035 20188146

[B59] Varadarajan S. Bampton E. T. Smalley J. L. Tanaka K. Caves R. E. Butterworth M.. (2012). A novel cellular stress response characterised by a rapid reorganisation of membranes of the endoplasmic reticulum. Cell Death Differ. 19, 1896–1907. 10.1038/cdd.2012.108 22955944 PMC3504701

[B60] Varadarajan S. Tanaka K. Smalley J. L. Bampton E. T. Pellecchia M. Dinsdale D.. (2013). Endoplasmic reticulum membrane reorganization is regulated by ionic homeostasis. PLoS ONE 8:e56603. 10.1371/journal.pone.0056603 23457590 PMC3574070

[B61] Voeltz G. K. Prinz W. A. Shibata Y. Rist J. M. Rapoport T. A. (2006). A class of membrane proteins shaping the tubular endoplasmic reticulum. Cell 124, 573–586. 10.1016/j.cell.2005.11.047 16469703

[B62] Wang S. Tukachinsky H. Romano F. B. Rapoport T. A. (2016). Cooperation of the ER-shaping proteins atlastin, lunapark, and reticulons to generate a tubular membrane network. Elife 5:e18605 10.7554/eLife.18605 27619977 PMC5021524

[B63] Wang T. Liu Y. Xu X. H. Deng C. Y. Wu K. Y. Zhu J.. (2011). Lgl1 activation of rab10 promotes axonal membrane trafficking underlying neuronal polarization. Dev. Cell 21, 431–444. 10.1016/j.devcel.2011.07.007 21856246

[B64] Westrate L. M. Lee J. E. Prinz W. A. Voeltz G. K. (2015). Form follows function: the importance of endoplasmic reticulum shape. Annu. Rev. Biochem. 84, 791–811. 10.1146/annurev-biochem-072711-163501 25580528

[B65] Wu Q. Sun X. Yue W. Lu T. Ruan Y. Chen T.. (2016). RAB18, a protein associated with Warburg Micro syndrome, controls neuronal migration in the developing cerebral cortex. Mol. Brain 9:19. 10.1186/s13041-016-0198-2 26879639 PMC4754921

[B66] Yamamoto A. Masaki R. Tashiro Y. (1996). Formation of crystalloid endoplasmic reticulum in COS cells upon overexpression of microsomal aldehyde dehydrogenase by cDNA transfection. J. Cell Sci. 109(Pt 7), 1727–1738. 8832395 10.1242/jcs.109.7.1727

[B67] Yamanaka T. Miyazaki H. Oyama F. Kurosawa M. Washizu C. Doi H.. (2008). Mutant Huntingtin reduces HSP70 expression through the sequestration of NF-Y transcription factor. EMBO J. 27, 827–839. 10.1038/emboj.2008.23 18288205 PMC2274932

[B68] Yamanaka T. Tosaki A. Kurosawa M. Matsumoto G. Koike M. Uchiyama Y.. (2014). NF-Y inactivation causes atypical neurodegeneration characterized by ubiquitin and p62 accumulation and endoplasmic reticulum disorganization. Nat. Commun. 5:3354. 10.1038/ncomms4354 24566496

[B69] Yamanaka T. Tosaki A. Miyazaki H. Kurosawa M. Koike M. Uchiyama Y.. (2016). Differential roles of NF-Y transcription factor in ER chaperone expression and neuronal maintenance in the CNS. Sci. Rep. 6:34575. 10.1038/srep34575 27687130 PMC5043352

[B70] Yang Y. S. Harel N. Y. Strittmatter S. M. (2009). Reticulon-4A (Nogo-A) redistributes protein disulfide isomerase to protect mice from SOD1-dependent amyotrophic lateral sclerosis. J. Neurosci. 29, 13850–13859. 10.1523/JNEUROSCI.2312-09.2009 19889996 PMC2797811

